# Repeatability and reproducibility of corneal higher-order aberrations measurements after small incision lenticule extraction using the Scheimpflug-Placido topographer

**DOI:** 10.1186/s40662-021-00274-y

**Published:** 2022-01-04

**Authors:** Rui Ning, Rongrong Gao, David P. Piñero, Jun Zhang, Qingyi Gao, Yili Jin, Yiran Wang, Chenxiao Wang, Jinhai Huang

**Affiliations:** 1 Eye Hospital and School of Ophthalmology and Optometry, Wenzhou Medical University, Wenzhou, Zhejiang China; 2 Eye Institute and Department of Ophthalmology, Eye and ENT Hospital, Fudan University, Shanghai, China; 3 Group of Optics and Visual Perception, Department of Optics, Pharmacology and Anatomy, University of Alicante, Alicante, Spain; 4 NHC Key Laboratory of Myopia (Fudan University), Key Laboratory of Myopia, Chinese Academy of Medical Sciences, Shanghai, China; 5 Shanghai Research Center of Ophthalmology and Optometry, Shanghai, China

**Keywords:** Sirius, Higher-order aberrations, Repeatability, Reproducibility, Small incision lenticule extraction

## Abstract

**Background:**

To evaluate the precision of corneal higher-order aberrations measurements after small incision lenticule extraction (SMILE) using the Sirius Scheimpflug-Placido topographer (CSO, Italy).

**Methods:**

Seventy-five eyes from 75 postoperative subjects were included in this prospective study. Three consecutive corneal aberrometric measurements were obtained with the Scheimpflug-Placido topographer by two experienced operators to assess intra- and inter-observer reproducibility. The within-subject standard deviation (S_w_), test-retest repeatability (TRT) and the intraclass correlation coefficient (ICC) were calculated.

**Results:**

For intraobserver repeatability of anterior and total corneal aberrations, all ICCs were more than 0.922, except for trefoil (0.722 to 0.768). The ICCs of total root mean square (RMS), coma Z (3, ± 1), and spherical aberration Z (4, 0) were over 0.810 while higher-order RMS, trefoil Z (3, ± 3), and astigmatism II Z (4, ± 2) were below 0.634 for posterior corneal surface aberrations. All S_w_ values for all types of aberrations were equal to or below 0.07 μm. Regarding interobserver reproducibility, all TRT values were no more than 0.12 μm, 0.05 μm, and 0.11 μm for anterior, posterior, and total corneal aberrations, respectively. The ICC values ranged from 0.875 to 0.989, from 0.686 to 0.976 and over 0.834 for anterior, posterior, and total corneal aberrations, respectively.

**Conclusions:**

The repeatability of measurements of anterior and total corneal aberrations with the Sirius system in corneas after SMILE surgery was high, except for trefoil. There was some variability in posterior corneal aberrometric measurements. High reproducibility of corneal aberrometric measurements was observed between measurements of both examiners, except for trefoil, with poor to moderate reproducibility.

## Background

Corneal refractive surgery has evolved over decades, with several techniques aimed at correcting ametropia by changing the curvature and shape of the corneal surface, along with associated changes in asphericity and high-order aberrations (HOAs) [1, 2]. After surgery, the improvement in vision can be accompanied by some visual effects, such as glare, halos, reduced contrast sensitivity, and poor night vision [3, 4]. Analysis of corneal aberrations is helpful to guide and predict the long-term visual quality after operation. An accurate and comprehensive understanding of corneal morphology is of great significance for preoperative screening, postoperative follow-up, and safety assessment of corneal refractive surgery.

The Sirius system (CSO, Firenze, Italy) is a tomographer for clinical use based on Scheimpflug imaging technology combined with Placido-based reflection. Several attempts have been made to certify the good intrasession repeatability and reproducibility of anterior segment measurements provided by this device [5–8]. The change of corneal morphology caused by refractive surgery and the increase of the severity of keratoconus might lead to the decrease of measurement repeatability and consistency for most of anterior segment parameters [9–12]. Jin et al. [9] reported that corneal refractive surgery changed the postoperative interdevice differences in corneal curvature measurements and reduced interdevice agreement of four different devices. It is therefore uncertain whether the accuracy of the aberration measurement associated to corneal irregularity is consistent with other anterior segment parameters. Ortiz et al. [13] indicated that the repeatability of the Zernike coefficients of corneal aberrometry tended to improve with increasing keratoconus stage using a Placido-disk device. Savini et al. [8] evaluated the repeatability of corneal spherical aberration measured with the Sirius system and found higher intraclass correlation coefficient (ICC) in eyes after photorefractive keratectomy (PRK) or laser in situ keratomileusis (LASIK) (ICC 0.980) and keratoconus eyes (ICC 0.981) than in normal eyes (ICC 0.806). Similar outcomes were presented by Bayhan et al. [14], obtaining higher ICCs of anterior and posterior corneal aberrations in keratoconus eyes compared with normal eyes. Despite these studies, there was a lack of research on the precision (repeatability and reproducibility) of corneal aberration measurement in patients after small incision lenticule extraction (SMILE). A change in HOAs of 0.05 μm can decrease visual acuity [15] although most previous studies on corneal aberrations have considered the anterior surface only. The SMILE surgical technique, as any other corneal refractive surgery procedure, alters the compensation mechanism for spherical aberration and HOAs between anterior and posterior surfaces of the cornea. Thus, an accurate analysis of corneal aberrations after SMILE cannot ignore the posterior surface. The aim of the current study was to assess the repeatability and interobserver reproducibility of Scheimpflug-Placido topography measurements of anterior, posterior, and total corneal aberrations after SMILE. To our knowledge, this is the first study to evaluate this issue.

## Methods

### Patient

The study was conducted at the Eye Hospital of Wenzhou Medical University, Wenzhou, China. All patients were informed in advance regarding the purpose of the study and signed an informed consent following the tenets of the Helsinki Declaration. Patients after SMILE were enrolled in our study. All patients recovered well after operation with stable refraction, and one eye of each patient was randomly selected for the study. The minimum time since surgery prior to inclusion was 3 months. Exclusion criteria were history of other intraocular surgeries or ocular trauma, severe dry eye, conjunctivitis, ocular infection and other corneal diseases, any active systemic disease, or the use of systemic drugs that may affect corneal wound healing.

### Surgical technique

One experienced surgeon performed all surgeries using the 500-kHz VisuMax femtosecond laser (Carl Zeiss Meditec, Jena, Germany). Surgeries were performed under topical anesthesia and patients were asked to fixate on a blinking light. The eyeball was fixed with a negative pressure suction ring, and the posterior lamellar surface of the refractive procedure was performed first. Next, a side-cut at the outer border of the lamellar dissection was performed. Finally, the cap scanned from the center to the periphery was started to make a 1.8–2.0 mm corneal incision. Then, the refractive lenticule of the intrastromal corneal tissue was extracted through the small side-cut. One drop of tobramycin dexamethasone and levofloxacin 0.5% was applied immediately after surgery.

### Topographic measurement procedure

To ensure the accuracy of the measurement, all measurements were carried out in a dim room, without induction of pharmacological mydriasis. Patients were instructed to sit in front of the instrument, and the seat height was adjusted to make the patient sit comfortably. Then, the mandible was properly placed on the mandibular support, with forehead contacting with the forehead support, and with an adjustment of the mandibular height to be aligned with the outer canthus line. Subjects were asked to fixate on the target of the instrument after full blinking. To evaluate the intraobserver repeatability and interobserver reproducibility, two skilled operators obtained three measurements in a random order. The quality of the acquired image was over the minimum percentage required by the instrument criteria: Scheimpflug image coverage ≥ 90%, Scheimpflug image reliability ≥ 90%, corneal projection center positioning ≥ 90%, and corneal projection coverage ≥ 85%.

### Topography measurement system

**Fig. 1:**
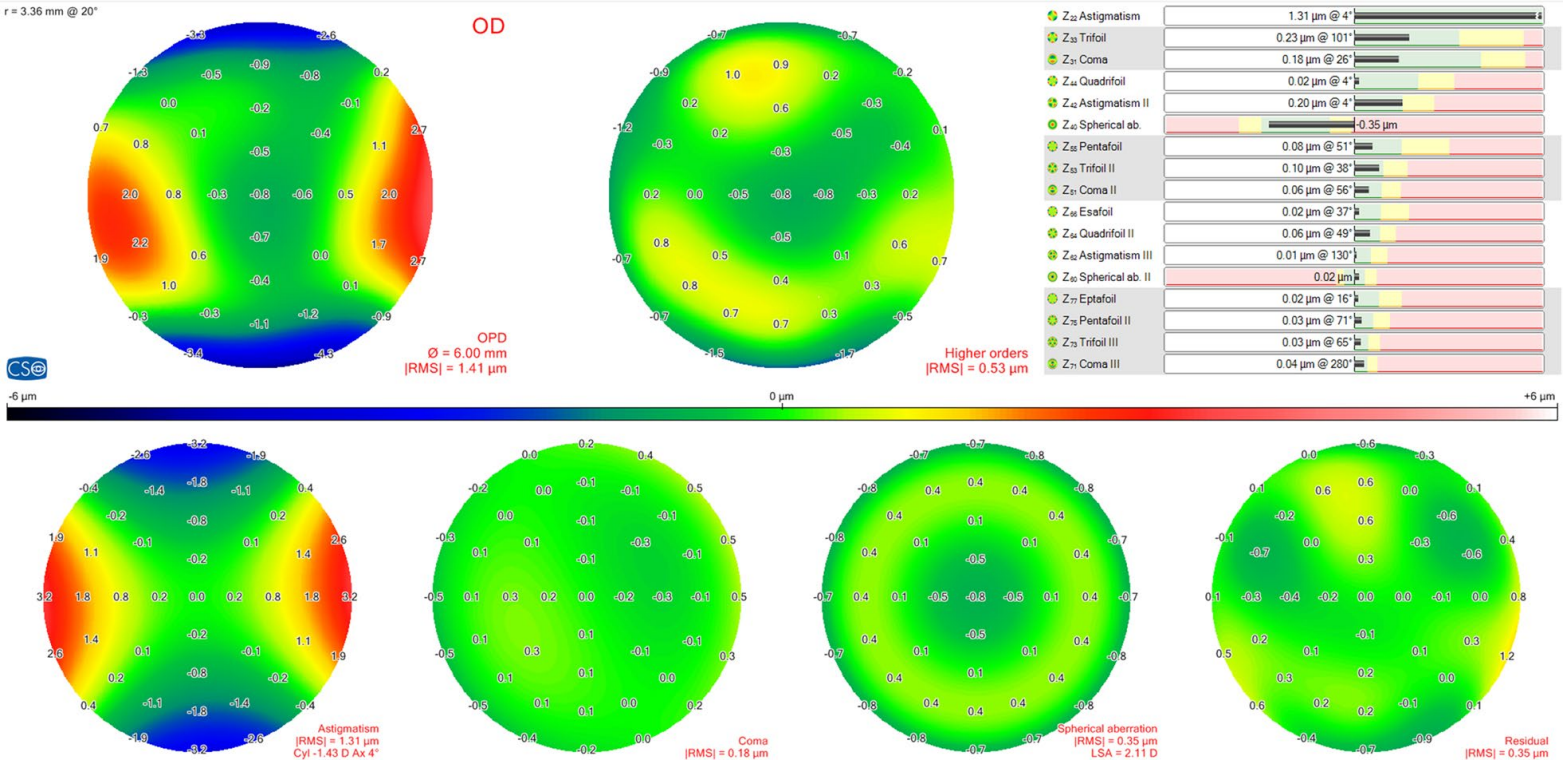
Wavefront aberration analysis output diagram

The Sirius system is a tomography device that combines two technologies, a Scheimpflug rotating camera and Placido-disk reflection topography. There are proprietary methods to merge the anterior surface data from Placido and Scheimpflug images. Data for the posterior corneal surface is derived only from Scheimpflug imaging. It enables rapid acquisition and processing of 25 radial sections of the cornea and anterior chamber, which provides curvature data of anterior and posterior corneal surfaces, corneal pachymetry, and corneal wavefront. The program associated with the device permits conducting an analysis of the wavefront aberrations generated by the cornea using Zernike polynomial analysis. In addition, this aberrometric analysis can be obtained for the total cornea (considering both anterior and posterior surfaces), for the anterior surface only and also for the posterior corneal surface [14]. Figure 1 shows the output of wavefront analysis provided by the Sirius device.

In this study, the following parameters were recorded and evaluated: astigmatism II Z (4, ± 2), trefoil Z (3, ± 3), coma Z (3, ± 1), spherical aberration Z (4, 0), high-order root mean square (RMS) and total RMS up to 7th order in Sirius system. All these parameters were calculated for a 6.0 mm diameter area of analysis [14, 16–19]. The unit of measure is microns.

### Statistical analysis

Statistical analysis was performed using SPSS software (version 21.0, IBM Corporation, USA). The intraobserver repeatability and interobserver reproducibility were assessed by means of the following statistical variables: within-subject standard deviation (S_w_), test-retest repeatability (TRT) and ICC. The S_w_ is a simple method of calculating the size of the measurement error. The TRT is obtained by multiplying S_w_ by 2.77, which represents the error range of repeated measurements in 95% of the observations [20]. The ICC is an analysis of variance-based type of correlation which represents the consistency of datasets of repeated measurements. The ICC values range from 0 to 1 and the closer the ICC is to 1, the better the measurement consistency is [21].

## Results

Seventy-five eyes of 75 patients (51 men) that had undergone SMILE surgery were included. Age of patients ranged from 18 to 41 years (23.35 ± 5.61 years). The preoperative mean spherical equivalent refraction was − 4.72 ± 1.51 diopters (D) (range − 8.625 to − 2.00 D). The postoperative uncorrected distance visual acuity at the moment where the experiment was conducted was − 0.08 ± 0.06 logMAR (range − 0.18 to 0.10 logMAR).

### Intraobserver repeatability

Tables 1, 2 and 3 show separately the intraobserver repeatability data corresponding to anterior, posterior, and total corneal aberrations, respectively. All S_w_ values for all types of aberrations were equal to or below 0.07 μm. For the anterior corneal aberrometric data (Table 1), the TRT values ranged from 0.06 to 0.19 μm, and ICC values were over 0.93, except for trefoil components (0.768 and 0.763). For the posterior corneal aberrometric data (Table 2), the S_w_ of each aberrometric parameter was less than 0.03 μm. The TRT values were below 0.09 μm and the ICCs ranged from 0.305 to 0.957, with values over 0.810 for total RMS, coma Z (3, ± 1), and spherical aberration Z (4, 0) coefficients, and values below 0.634 for high-order RMS, trefoil Z (3, ± 3), and astigmatism II Z (4, ± 2) coefficients. Regarding total corneal aberrations (Table 3), the ICC for the trefoil component Z (3, ± 3) had the lowest value, with the rest of ICCs being of more than 0.922. The TRT values were always below 0.19 μm.

**Table 1:** Intraobserver repeatability outcomes for anterior corneal aberrations

Parameter	Operator	Mean ± SD (μm)	S_w_ (μm)	TRT (μm)	ICC (95% CI)
Total RMS	1st	1.27 ± 0.42	0.05	0.14	0.985 (0.979–0.990)
	2nd	1.29 ± 0.42	0.07	0.19	0.973 (0.961–0.982)
Higher-order RMS	1st	0.63 ± 0.18	0.03	0.10	0.964 (0.948–0.976)
	2nd	0.64 ± 0.18	0.04	0.10	0.960 (0.942–0.973)
Coma Z (3, ± 1)	1st	0.36 ± 0.20	0.04	0.10	0.967 (0.952–0.978)
	2nd	0.36 ± 0.20	0.04	0.10	0.967 (0.952–0.978)
Trefoil Z (3, ± 3)	1st	0.17 ± 0.08	0.04	0.12	0.768 (0.682–0.838)
	2nd	0.19 ± 0.09	0.05	0.13	0.763 (0.676–0.834)
SA Z (4, 0)	1st	0.40 ± 0.12	0.02	0.06	0.969 (0.955–0.980)
	2nd	0.40 ± 0.12	0.02	0.06	0.967 (0.953–0.978)
Ast II Z (4, ± 2)	1st	0.13 ± 0.09	0.02	0.06	0.951 (0.929–0.967)
	2nd	0.14 ± 0.09	0.02	0.07	0.931 (0.900–0.953)

*SD* = standard deviation; *S*_*w*_ = within-subject standard deviation; *TRT* = test-retest repeatability (2.77 S_w_); *ICC* = intraclass correlation coefficient; *RMS* = root mean square; *SA* = spherical aberration; *Ast* = astigmatism

**Table 2:** Intraobserver repeatability outcomes for posterior corneal aberrations

Parameter	Operator	Mean ± SD (μm)	S_w_ (μm)	TRT (μm)	ICC (95% CI)
Total RMS	1st	0.36 ± 0.11	0.02	0.06	0.957 (0.938–0.971)
	2nd	0.36 ± 0.11	0.02	0.06	0.953 (0.932–0.968)
Higher-order RMS	1st	0.11 ± 0.03	0.02	0.07	0.597 (0.474–0.706)
	2nd	0.11 ± 0.03	0.03	0.07	0.526 (0.394–0.649)
Coma Z (3, ± 1)	1st	0.03 ± 0.02	0.01	0.02	0.927 (0.895–0.950)
	2nd	0.03 ± 0.02	0.01	0.02	0.892 (0.847–0.927)
Trefoil Z (3, ± 3)	1st	0.06 ± 0.03	0.03	0.08	0.432 (0.292–0.569)
	2nd	0.06 ± 0.03	0.03	0.09	0.305 (0.161–0.455)
SA Z (4, 0)	1st	0.02 ± 0.02	0.01	0.02	0.810 (0.738–0.869)
	2nd	0.02 ± 0.01	0.01	0.02	0.853 (0.794–0.899)
Ast II Z (4, ± 2)	1st	0.02 ± 0.01	0.01	0.02	0.634 (0.518–0.735)
	2nd	0.02 ± 0.01	0.01	0.02	0.585 (0.461–0.697)

*SD* = standard deviation; *S*_*w*_ = within-subject standard deviation; *TRT* = test-retest repeatability (2.77 S_w_); *ICC* = intraclass correlation coefficient; *RMS* = root mean square; *SA* = spherical aberration; *Ast* = astigmatism

**Table 3:** Intraobserver repeatability outcomes for total corneal aberrations

Parameter	Operator	Mean ± SD (μm)	S_w_ (μm)	TRT (μm)	ICC (95% CI)
Total RMS	1st	1.01 ± 0.33	0.05	0.14	0.979 (0.969–0.986)
	2nd	1.03 ± 0.33	0.07	0.19	0.957 (0.937–0.971)
Higher-order RMS	1st	0.62 ± 0.18	0.03	0.10	0.964 (0.947–0.976)
	2nd	0.63 ± 0.18	0.04	0.11	0.953 (0.932–0.968)
Coma Z (3, ± 1)	1st	0.35 ± 0.19	0.04	0.10	0.965 (0.949–0.977)
	2nd	0.34 ± 0.20	0.04	0.12	0.955 (0.935–0.970)
Trefoil Z (3, ± 3)	1st	0.17 ± 0.09	0.05	0.14	0.722 (0.624–0.803)
	2nd	0.19 ± 0.09	0.05	0.15	0.722 (0.625–0.803)
SA Z (4, 0)	1st	0.38 ± 0.12	0.02	0.06	0.967 (0.953–0.978)
	2nd	0.38 ± 0.12	0.02	0.07	0.964 (0.948–0.976)
Ast II Z (4, ± 2)	1st	0.14 ± 0.09	0.02	0.06	0.944 (0.919–0.962)
	2nd	0.14 ± 0.09	0.03	0.07	0.922 (0.888–0.947)

*SD* = standard deviation; *S*_*w*_ = within-subject standard deviation; *TRT* = test-retest repeatability (2.77 S_w_); *ICC* = intraclass correlation coefficient; *RMS* = root mean square; *SA* = spherical aberration; *Ast* = astigmatism

### Interobserver reproducibility

Tables 4, 5, 6 display mean values, S_w_, TRT and ICCs of the corneal aberrometric data obtained by the two observers. All TRT values of interobserver reproducibility were excellent because of their low variability, being no more than 0.12 μm, 0.05 μm, and 0.11 μm for anterior, posterior, and total corneal aberrometric data, respectively. The ICC values ranged from 0.875 to 0.989 for anterior corneal aberrations, and 0.686 to 0.976 for posterior corneal aberrations. The analysis of the interobserver reproducibility of total corneal aberrations showed that most of ICCs were over 0.979, except for trefoil (0.834).

**Table 4:** Interobserver reproducibility for anterior corneal aberrations between the two observers

Parameter	Mean ± SD (μm)	S_w_ (μm)	TRT (μm)	ICC (95% CI)
Total RMS	1.28 ± 0.42	0.04	0.12	0.989 (0.982–0.993)
Higher-order RMS	0.64 ± 0.18	0.02	0.07	0.983 (0.973–0.989)
Coma Z (3, ± 1)	0.36 ± 0.20	0.03	0.07	0.984 (0.974–0.990)
Trefoil Z (3, ± 3)	0.18 ± 0.08	0.03	0.08	0.875 (0.802–0.921)
SA Z (4, 0)	0.40 ± 0.12	0.01	0.03	0.989 (0.983–0.993)
Ast II Z (4, ± 2)	0.13 ± 0.09	0.01	0.04	0.974 (0.960–0.984)

*SD* = standard deviation; *S*_*w*_ = within-subject standard deviation; *TRT* = test-retest repeatability (2.77 S_w_); *ICC* = intraclass correlation coefficient; *RMS* = root mean square; *SA* = spherical aberration; *Ast* = astigmatism

**Table 5:** Interobserver reproducibility for posterior corneal aberrations between the two observers

Parameter	Mean ± SD (μm)	S_w_ (μm)	TRT (μm)	ICC (95% CI)
Total RMS	0.36 ± 0.11	0.02	0.05	0.976 (0.962–0.985)
Higher-order RMS	0.11 ± 0.03	0.01	0.04	0.845 (0.766–0.899)
Coma Z (3, ± 1)	0.03 ± 0.02	0.00	0.01	0.967 (0.948–0.979)
Trefoil Z (3, ± 3)	0.06 ± 0.03	0.02	0.05	0.686 (0.544–0.789)
SA Z (4, 0)	0.02 ± 0.02	0.01	0.01	0.886 (0.826–0.926)
Ast II Z (4, ± 2)	0.02 ± 0.01	0.00	0.01	0.819 (0.728–0.882)

*SD* = standard deviation; *S*_*w*_ = within-subject standard deviation; *TRT* = test-retest repeatability (2.77 S_w_); *ICC* = intraclass correlation coefficient; *RMS* = root mean square; *SA* = spherical aberration; *Ast* = astigmatism

**Table 6:** Interobserver reproducibility for total corneal aberrations between the two observers

Parameter	Mean ± SD (μm)	S_w_ (μm)	TRT (μm)	ICC (95% CI)
Total RMS	1.02 ± 0.33	0.04	0.11	0.987 (0.978–0.992)
Higher-order RMS	0.62 ± 0.18	0.02	0.07	0.981 (0.970–0.988)
Coma Z (3, ± 1)	0.35 ± 0.20	0.03	0.08	0.979 (0.968–0.987)
Trefoil Z (3, ± 3)	0.18 ± 0.09	0.04	0.10	0.834 (0.744–0.893)
SA Z (4, 0)	0.38 ± 0.12	0.01	0.04	0.989 (0.983–0.993)
Ast II Z (4, ± 2)	0.14 ± 0.09	0.02	0.04	0.985 (0.954–0.982)

*SD* = standard deviation; *S*_*w*_ = within-subject standard deviation; *TRT* = test-retest repeatability (2.77 S_w_); *ICC* = intraclass correlation coefficient; *RMS* = root mean square; *SA* = spherical aberration; *Ast* = astigmatism

## Discussion

The demand of improvement for visual quality has gained importance in step with the advances in refractive surgery technology. However, laser refractive surgery to eliminate low refractive errors could cause irregular changes of the corneal surface, with the induction of HOAs, which adversely affects the postoperative visual function [22–24]. Several studies [25–27] have demonstrated that total HOAs, coma and spherical aberration increase significantly after SMILE procedures. For this reason, accurate HOAs measurement is essential for evaluating the potential impact of surgery in visual quality. Previous studies [5–7] have already reported good to excellent intrasession repeatability and reproducibility of anterior segment measurements obtained with the Sirius system in healthy, post-refractive surgery (PRK/LASIK) and keratoconus patients. Our study enrolled 75 eyes that had undergone laser corneal refractive surgery with another type of technique, SMILE surgery. To explore the precision of corneal morphology measurements in more detail and comprehensively, this study was designed to evaluate the aberrations of the anterior and posterior surface as well as the total cornea with Sirius Scheimpflug-Placido topographer.

### Intraobserver repeatability

In this study, the repeatability of anterior corneal aberrations was excellent, with ICCs for all types of aberrations being more than 0.931 and S_w_ values being lower than 0.07 μm, except for trefoil. In any case, the level of repeatability for trefoil components was also acceptable, with ICC of 0.768 for the first operator and 0.763 for the second operator. Bayhan et al. [14] found the similar results with the Sirius device, obtaining good repeatability for anterior corneal aberrations in normal eyes, with ICCs ranging from 0.678 to 0.976 for astigmatism II Z (4, ± 2), trefoil Z (3, ± 3), coma Z (3, ± 1), spherical aberration Z (4, 0), higher-order RMS and total RMS. The only difference was that S_w_ values of Zernike coefficients corresponding to astigmatism II were lower (0.02 μm) in our study. As with previous studies [13, 14, 28] assessing the consistency of corneal spherical aberration measurements in healthy eyes, the repeatability associated to this Zernike coefficient was higher than that corresponding to other aberrometric components. The mean value of corneal high-order RMS was 0.63 ± 0.18 μm here, which was a higher magnitude than that reported in a previous study for healthy eyes (0.41 ± 0.06 μm) using the same Scheimpflug-Placido topographer [14]. As expected according to previous literature, HOAs of the anterior corneal surface are increased after SMILE.

The difference in refractive index between the cornea and the aqueous humor is approximately 10% of the difference of indexes between cornea and air. It is, therefore, likely that the anterior corneal surface makes a significant contribution to ocular aberrations [29–31]. Because of this, it was expected that ICCs of total corneal aberrations were similar to those corresponding to the anterior surface (Table 3). The S_w_ of spherical aberration was the lowest (0.02 μm) in our study for both anterior and total corneal calculations. Then, it can be considered as an optical effective reference, especially for those surgeons that implant aspheric IOLs [32]. The trefoil Z (3, ± 3) components showed the worst ICC value (0.722) and a high S_w_ (0.14 μm). Similar results were reported by Aramberri et al. [18] and Cervino et al. [17], who investigated the repeatability and reproducibility of the Scheimpflug imaging-based Pentacam and Galilei systems in healthy eyes. They found moderate to high precision in coma Z (3, ± 1) and spherical aberration Z (4, 0) coefficients (ICC = 0.74–0.915), with the worst results for trefoil Z (3, ± 3) (ICCs of 0.478 and 0.552). Cervino et al. [17] indicated that the reason for this variability might be the incomplete eye opening during the measurement procedure, causing the outliers of superior periphery, which especially affected the 90-degree coma coefficient and both trefoil coefficients. Our results also demonstrated that the total RMS had the highest ICC value associated (0.979). The mean total corneal high-order RMS obtained with the Scheimpflug-Placido device was 0.63 ± 0.18 μm in our study, a value close to that reported by Ventura et al. [33] (0.63 ± 0.77 μm) using Placido topographer and lower than that obtained (0.93 ± 0.37 μm) using the dual Scheimpflug device in post-refractive surgery (PRK/LASIK) eyes.

Compared to the previous studies in healthy eyes [17, 18], our results showed better repeatability. The increase within acceptable levels of aberrations and irregularity of the cornea after the laser refractive surgery procedure may account for this better repeatability. These trends were in agreement with other studies [13, 14, 34], in which the results also showed better repeatability in irregular corneas compared to healthy eyes. In one study performing the repeatability analysis using keratoconus eyes, the authors concluded that the magnitude of HOAs were higher and could be clearly defined, being this a possible cause of more repeatable and useful measurements for the disease classification [13].

A previous study by Li et al. [35] indicated that the posterior corneal surface played an important role in compensating for the spherical aberration of the anterior surface of the cornea. There were few reports on repeatability of posterior corneal aberrometric measurements to this date [19, 34]. None of them have enrolled patients after SMILE. In our study, the S_w_ corresponding to each aberration of the posterior corneal surface was below 0.03 μm. The ICCs ranged from 0.432 to 0.957 for the first operator and from 0.305 to 0.953 for the second operator. Good precision values were only found for total RMS, coma Z (3, ± 1) and spherical aberration Z (4, 0). However, for high-order RMS, trefoil Z (3, ± 3) and astigmatism II Z (4, ± 2), more limited ICCs were obtained. In general, the central coefficients of the Zernike pyramidal diagram showed better intraobserver repeatability. The ICCs for coma (0.892–0.927) were better than those corresponding to trefoil (0.305–0.432) in the 3rd order and ICCs for spherical aberration (0.810–0.853) were better than those corresponding to astigmatism II (0.585–0.634) in the 4th order. Similar outcomes were also presented in former studies. In a study conducted by Bayhan et al. [14], the ICCs for posterior corneal coma (0.824) and spherical aberration (0.822) were better than those corresponding to trefoil (0.802) and astigmatism II (0.691) in healthy eyes. Similar results were also found in the study by Piñero et al. [19], in which the repeatability of Zernike coefficients was better for the aberrometric defects of the center of the Zernike pyramid than for those corresponding to the periphery of the Zernike pyramid at the 3rd and 4th order when using the Pentacam Scheimpflug imaging system in normal eyes (ICC > 0.943 for coma and spherical aberration, ICC < 0.887 for astigmatism II and trefoil). De Jong et al. [28] also reported that the Galilei G2 and Pentacam HR instruments provided similar and good repeatability in measuring the posterior corneal shape, except for oblique astigmatism and the two trefoil terms in the healthy eye. Furthermore, these results suggested that the ICCs of the Zernike coefficients describing the posterior corneal aberrations were lower compared with their anterior counterparts when comparing with the anterior corneal aberrations in our current study. Although the S_w_ and TRT values were lower for posterior corneal aberrometric data than those corresponding to the anterior surface, differences may be of little clinical relevance due to the small magnitude of such variability. In any case, one reason for this higher variability of posterior corneal aberrometric measurements may be in relation to the significantly lower magnitude of these aberrations compared to the anterior corneal surface, representing any small change that resulting in a significant level of variability. Additionally, the reason for the poor repeatability of posterior corneal aberrometric data might be the inadequate characterization of posterior corneal aberrations with Zernike polynomial expansion due to subtle movements during scanning [14]. More studies are still needed to overcome these potential limitations.

### Interobserver reproducibility

Regarding the reproducibility results of the corneal aberrometric components analyzed by both observers in our study, all S_w_ values for all types of aberrations were equal to or below 0.04 μm. The reliability analysis showed ICCs ranging from 0.834 to 0.989 for anterior and total corneal aberrations, with the lowest ICC value for the trefoil component Z (3, ± 3), suggesting excellent interobserver reproducibility except for trefoil. Concerning posterior corneal aberrometric data, ICCs of total RMS and coma Z (3, ± 1) were 0.976 and 0.967, also indicating excellent interobserver reproducibility. These results were better than those from a previous study conducted by Sideroudi et al. [34] evaluating the reproducibility of Pentacam-derived posterior aberrations measurements in both normal and ectatic corneas, with coma, coma-like and HOA RMS showing acceptable reliability. Moreover, the excellent interobserver reproducibility observed in our series was consistent with that previously reported for other anterior segment parameters [7, 36]. Hernandez et al. [7] analyzed the cornea and anterior segment using the Sirius system, obtaining ICC values of more than 0.9 for all of the measured variables. Bao et al. [36] who evaluated the reproducibility of posterior corneal surface measurement using the Sirius system also reported high reproducibility for posterior corneal surface measurement in normal eyes. To our knowledge, this is the first report that evaluates the reproducibility of Sirius measurements of corneal aberrations in post-SMILE eyes.

It should be noted that there are some factors that may account for the significantly decreased precision, such as misalignment or movement during scanning, short acquisition time, pupil translation [37], and tear film instability. Current results indicate that the repeatability and reproducibility of trefoil were the worst. The reason might be incomplete eye opening during the measurement procedure, which limited acquisition of the superior cornea. Certain instruments with different principles show a similar phenomenon. Previous studies found similar results with the Scheimpflug imaging-based topographer [14], new pyramid wavefront sensor [38], and Hartmann-Shack aberrometer [39]. We also acknowledge that patients included in this study were young and well-coordinated, with a successful recovery after SMILE without complications. Therefore, further research may include patients with a wider age range, and evaluate which device can more precisely determine the aberrations of the posterior surface of the cornea.

## Conclusions

In conclusion, the repeatability of anterior corneal aberrations and total corneal aberrations was high, with a moderate repeatability for the trefoil component. Likewise, the Sirius system showed some variability in a few of the posterior corneal aberrations parameters. In terms of reproducibility, all corneal aberrations showed low variability between measurements of both examiners, except for trefoil, which had poor to moderate reproducibility. These findings suggest that the Scheimpflug-Placido can be used to reliably quantify the HOAs of the anterior and total cornea. The measurement of posterior corneal aberrations although less consistent, may still be considered for clinical use but with some range of variability.

## Data Availability

All data analyzed during this study are included in this published article.

## Abbreviations

ICC: Intraclass correlation coefficient
PRK: Photorefractive keratectomy
LASIK: Laser in situ keratomileusis
SMILE: Small incision lenticule extraction
RMS: Root mean square
S_w_: Standard deviation
TRT: Test-retest repeatability
HOAs: High-order aberrations
